# Melatonin-Mediated Abiotic Stress Tolerance in Plants

**DOI:** 10.3389/fpls.2022.847175

**Published:** 2022-05-09

**Authors:** Wen Zeng, Salma Mostafa, Zhaogeng Lu, Biao Jin

**Affiliations:** ^1^College of Horticulture and Plant Protection, Yangzhou University, Yangzhou, China; ^2^Department of Floriculture, Faculty of Agriculture, Alexandria University, Alexandria, Egypt

**Keywords:** melatonin, abiotic stress, stress tolerance, exogenous applications, biosynthesis, metabolism, antioxidants, molecular signaling

## Abstract

Melatonin is a multi-functional molecule that is ubiquitous in all living organisms. Melatonin performs essential roles in plant stress tolerance; its application can reduce the harmful effects of abiotic stresses. Plant melatonin biosynthesis, which usually occurs within chloroplasts, and its related metabolic pathways have been extensively characterized. Melatonin regulates plant stress responses by directly inhibiting the accumulation of reactive oxygen and nitrogen species, and by indirectly affecting stress response pathways. In this review, we summarize recent research concerning melatonin biosynthesis, metabolism, and antioxidation; we focus on melatonin-mediated tolerance to abiotic stresses including drought, waterlogging, salt, heat, cold, heavy metal toxicity, light and others. We also examine exogenous melatonin treatment in plants under abiotic stress. Finally, we discuss future perspectives in melatonin research and its applications in plants.

## Introduction

Plants encounter various environmental stresses throughout their lives. As sessile organisms, plants have evolved multiple response mechanisms to cope with adverse conditions, thus ensuring their survival and reproductive success. N-acetyl-5-methoxytryptamine (melatonin), an indolic compound derived from tryptophan, is a universal abiotic stress regulator in plants (Wang et al., 2018). Melatonin was first identified in the bovine pineal gland in 1958 (Lerner et al., 1958). It was later characterized as an essential animal hormone that is involved in multiple biological processes, including antioxidation, circadian rhythms, seasonal reproduction, sleep, sexual behavior, mood, temperature homeostasis, retina physiology, and immunological enhancement (Lerner et al., 1958; Shi et al., 2015b). In 1995, melatonin was first identified in plants (Dubbels et al., 1995; Hattori et al., 1995). Since then, melatonin has been shown to participate in various abiotic stress responses as a pleiotropic signaling molecule. In addition, it is an efficient scavenger of both reactive oxygen species (ROS) and reactive nitrogen species (RNS; Arnao and Hernández-Ruiz, 2019b). Melatonin is present in the leaves, roots, stems, petals, flower buds, fruits and seeds of various plant species. Its biosynthesis typically occurs in chloroplasts and mitochondria (Tan and Reiter, 2020).

Since the discovery of melatonin, numerous studies have been conducted to examine its functions in plants, thus revealing its protective roles against both biotic and abiotic stresses. In adverse environments, melatonin regulates plant growth and development by promoting seed germination, boosting lateral root generation, controlling flowering time and delaying leaf senescence (Arnao and Hernández-Ruiz, 2019a, 2020). Melatonin acts as a direct regulator by scavenging ROS and RNS. It also acts as an indirect regulator by regulating gene expression *via* stress-responsive transcription factors. Melatonin also functions as an auxin-like regulator, sharing a precursor molecule with auxin. It can mimic auxin activity, acting upstream of the auxin pathway to alter the expression profiles of various auxin-related transcription factors (e.g., WRKY, NAC, MYB, bHLH, and HD-ZIP family transcription factors; Liang et al., 2017; Tan and Reiter, 2020). Melatonin may also interact with other plant hormones such as indoleacetic acid (IAA), gibberellic acid (GA), cytokinin (CK), abscisic acid (ABA), ethylene (ET), salicylic acid (SA), jasmonic acid (JA), brassinosteroid (BR), strigolactones, and polyamines (Arnao and Hernández-Ruiz, 2020). Moreover, the exogenous application of melatonin can enhance plant tolerance (e.g., to drought, salt, heat, cold, waterlogging, and heavy metal toxicity) by modulating the biosynthesis of endogenous melatonin and the activities of antioxidative enzymes (Weeda et al., 2014; Zhang et al., 2015; Moustafa-Farag et al., 2020; Sun et al., 2021).

Considering its protective functions, melatonin has attracted increasing research attention in recent years because of the increasing harmful effects of climate change, soil salinization, and industrial pollution on agriculture, crop production, and food chain security. Over the past decade, considerable progress has been made in the general understanding of melatonin in plants. Therefore, an updated review highlighting recent findings in melatonin-mediated abiotic stress tolerance is needed. In this review, we have summarized the biosynthetic and catabolic pathways of melatonin. We have also focused on the defensive roles of melatonin against various abiotic stresses, as well as its applications for improving plant stress tolerance.

## Biosynthesis and Metabolism of Phytomelatonin

### Biosynthetic Pathway

Melatonin, which is ubiquitous among most plant species, has a low molecular weight and a stable structure (Wu Y. et al., 2021). Its biosynthetic pathways in model animals and plants have been elucidated. In animals, four main enzymes participate in the classic melatonin biosynthetic pathway. First, tryptophan hydroxylase converts tryptophan into 5-hydroxytryptophan, which is then decarboxylated by aromatic amino acid decarboxylase to form 5-hydroxytryptamine (serotonin). Next, arylalkylamine N-acetyltransferase, also known as serotonin N-acetyltransferase (SNAT), acetylates serotonin to form N-acetyl-5-hydroxytryptamine (N-acetylserotonin). Finally, hydroxyindole-O-methyltransferase, also known as N-acetylserotonin methyltransferase (ASMT), O-methylates N-acetyl-5-hydroxytryptamine to generate melatonin (Axelrod and Weissbach, 1960; Weissbach et al., 1960; Tan et al., 2016).

**Figure 1:**
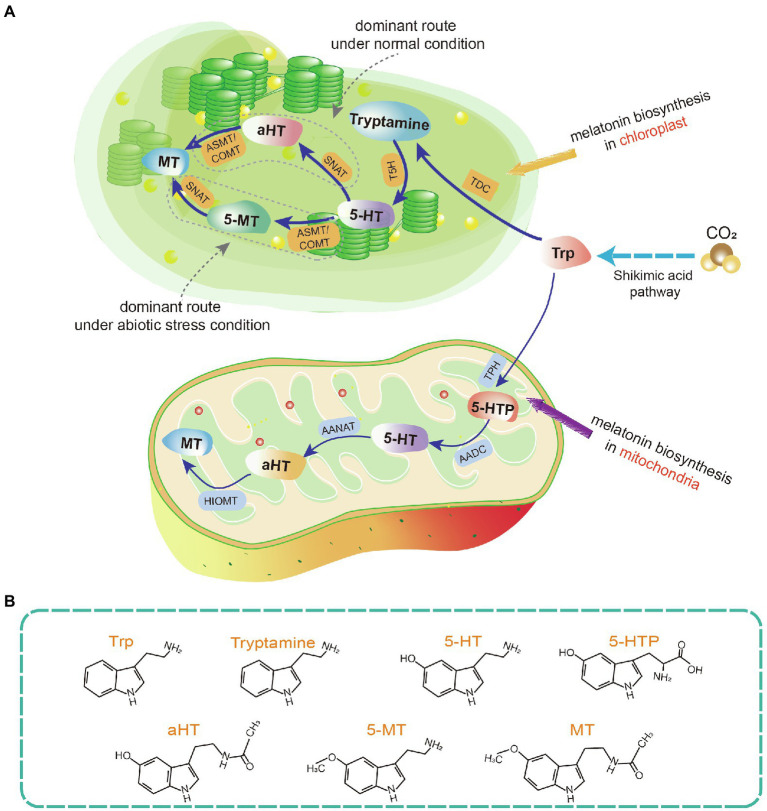
Phytomelatonin biosynthetic pathway in chloroplasts and mitochondria. **(A)** Schematic of melatonin biosynthetic pathways. **(B)** Molecular structures of key intermediate products in the melatonin biosynthetic pathway. Plants usually synthesize melatonin in chloroplasts under normal conditions. If this pathway is blocked, melatonin biosynthesis may be switched to the mitochondria. Trp, tryptophan; 5-HT, 5-hydroxytryptamine; 5-HTP, 5-hydroxytryptophan; aHT, N-acetyl-5-hydroxytryptamine; 5-MT, 5-methoxytryptamine; MT, melatonin; TDC, tryptophan decarboxylase; T5H, tryptamine 5-hydroxylase; SNAT, serotonin N-acetyltransferase; ASMT, N-acetylserotonin-O-methyltransferase (plant type SNATs and ASMTs appear to have origins distinct from the origins in animals); COMT, caffeic acid O-methyltransferase; TPH, tryptophan hydroxylase; AADC, aromatic amino acid decarboxylase; AANAT, arylalkylamine N-acetyltransferase (also known as arylamine N-acetyltransferase); and HIOMT, hydroxyindole-O-methyltransferase (also known as N-acetylserotonin O-methyltransferase).

The melatonin biosynthetic pathway in plants differs from the pathway in animals. It involves at least six biosynthetic enzymes including tryptophan decarboxylase (TDC), tryptophan hydroxylase, tryptamine 5-hydroxylase (T5H), SNAT, ASMT, and caffeic acid O-methyltransferase (COMT; Figure 1; Back et al., 2016; Sun et al., 2021). Among them, SNAT is a key rate-limiting enzyme (Liao et al., 2021). SNAT plant knockout strains exhibit altered phenotypes and become more sensitive to some abiotic stresses (Lee et al., 2019). ASMT and COMT were likely involved in plant terrestrialization. COMT first appeared in bryophytes and is presume to have evolved from ASMT. The increased activity of COMT compared to ASMT may have enhanced melatonin production in plants. In higher plants, COMT acquired an additional function in lignin biosynthesis (Zhao et al., 2021). Moreover, other hormonal pathways influence the expression of key melatonin biosynthesis genes. For example, ET insensitive protein 3 (*CcEIN3*) activates the expression of *CcTDC* and *CcASMT1* in hickory (Chen W. et al., 2021).

The first two biosynthesis steps in the plant melatonin pathway are the reverse of the first two steps in animals. In plants, tryptophan is decarboxylated by TDC in the first step, after which tryptamine is hydroxylated by T5H (Tan et al., 2016). SNAT and ASMT/COMT catalyze the final two biosynthesis steps, the order of which alternates depending on the environmental conditions (Byeon et al., 2015a). Under abiotic stress, the expression of different ASMT isoforms is induced, so serotonin is first O-methylated to form 5-methoxytryptamine by ASMT, and then acetylated to form melatonin (Ye et al., 2019; Tan and Reiter, 2020). In contrast, under standard conditions, the dominant pathway involves serotonin acetylation to form N-acetyl-5-hydroxytryptamine and then O-methylation to form melatonin (Ye et al., 2019; Figure 1).

In animals, melatonin is biosynthesized in the pineal gland. The specific organs in which melatonin is synthesized in plants are currently unknown. However, melatonin can be transported from roots to shoots as a long-distance signal in some plant species (Mukherjee et al., 2014; Li H. et al., 2017). The cellular location of melatonin biosynthesis in plants is clear. Tan et al. (2013) hypothesized that mitochondria and chloroplasts are the sites of melatonin biosynthesis in plants. The key melatonin synthetase, SNAT, is mainly localized to the chloroplasts in plants such as rice, cucumber, and tomato (Byeon et al., 2014; Wang et al., 2020). COMT and ASMT can be highly expressed in the chloroplast and their overexpression results in enhanced melatonin production (Choi et al., 2017). Taken together, this evidence suggests that the chloroplast may be the main site of melatonin biosynthesis. In addition to chloroplasts, mitochondria are regarded as sites of melatonin biosynthesis. The apple *MzSNAT5* gene, which is localized to mitochondria, is more closely related to animal SNAT than to the chloroplast-localized *MzSNAT9* or SNATs present in rice, *Arabidopsis*, or cyanobacteria (Wang et al., 2017; Tan and Reiter, 2020).

Mitochondria and chloroplasts are the biosynthetic sites of melatonin because of their evolutionary histories Hardeland, 2019. Melatonin is produced by several bacterial taxa, including cyanobacteria and α-proteobacteria. Mitochondria evolved from ingested α-proteobacteria, while chloroplasts originated from photosynthetic cyanobacteria. Throughout evolution, both organelles likely retained their ability to produce melatonin (Zhao et al., 2019). Moreover, there is evidence to suggest that plants preferentially perform melatonin biosynthesis in chloroplasts under normal conditions. When the chloroplast pathway is blocked, melatonin biosynthesis takes place in the mitochondria (Tan and Reiter, 2020; Figure 1).

### Metabolic Pathway

Melatonin can be degraded *via* enzymatic and non-enzymatic transformation routes (Tan et al., 2012; Hardeland, 2015; Back, 2021). In the enzymatic transformation route of animals, melatonin is metabolized into 6-hydroxymelatonin (6-OHM) by P450 enzymes. 6-OHM is then sulfated into 6-sulfatoxymelatonin and N1-acetyl-N2-formyl-5-methoxykynuramine (AFMK) by several enzymes (e.g., indoleamine 2,3-dioxygenase; Ma et al., 2005; Semak et al., 2005; Hardeland, 2017). 6-OHM is the major product of enzymatic melatonin degradation in animals. In non-enzymatic transformation routes, melatonin is degraded by free radicals or other oxidants. Examples of melatonin metabolites include cyclic 3-OHM (c3-OHM), 4-OHM, 2-OHM, AFMK, and N-acetyl-5-methoxykynuramine, which are produced non-enzymatically through interactions with ROS and RNS (Hardeland, 2017; Back, 2021).

The enzymatic degradation of melatonin in plants shares some common features with such degradation in vertebrates and other eukaryotes (Sun et al., 2021). For example, indoleamine 2,3-dioxygenase, which converts melatonin to AFMK, was identified in water hyacinth (*Eichhornia crassipes*) and other phototrophs such as phaeophytes, dinoflagellates, and chlorophyceae (Tan et al., 2007; Hardeland et al., 2009; Okazaki et al., 2010; Hardeland, 2015). Similarly, 2-OHM and c3-OHM are two melatonin hydroxylation metabolites present in the plant metabolic pathway. They are generated by melatonin 2-hydroxylase (M2H) and melatonin 3-hydroxylase (M3H), respectively (Byeon and Back, 2015; Byeon et al., 2015c; Lee et al., 2016; Choi and Back, 2019). Notably, M2H-1, a 2-oxoglutarate-dependent dioxygenase (2-ODD) isogene in rice, is localized to chloroplasts and has a major role in 2-OHM biosynthesis. Other 2-ODD isogenes with moderate M2H activity are expressed in the cytoplasm. The M2H pathway is the most important catabolic pathway, with crucial roles in modulating physiological and biochemical processes under biotic and abiotic stresses (Byeon et al., 2015b; Sun et al., 2021). There is evidence that endogenously triggered 2-OHM production, as well as exogenously applied 2-OHM, can improve plant tolerance to various adverse stresses such as cold, drought, metal stress, and pathogen attack (Lee and Back, 2016a; Shah et al., 2020; Tan and Reiter, 2020).

**Figure 2:**
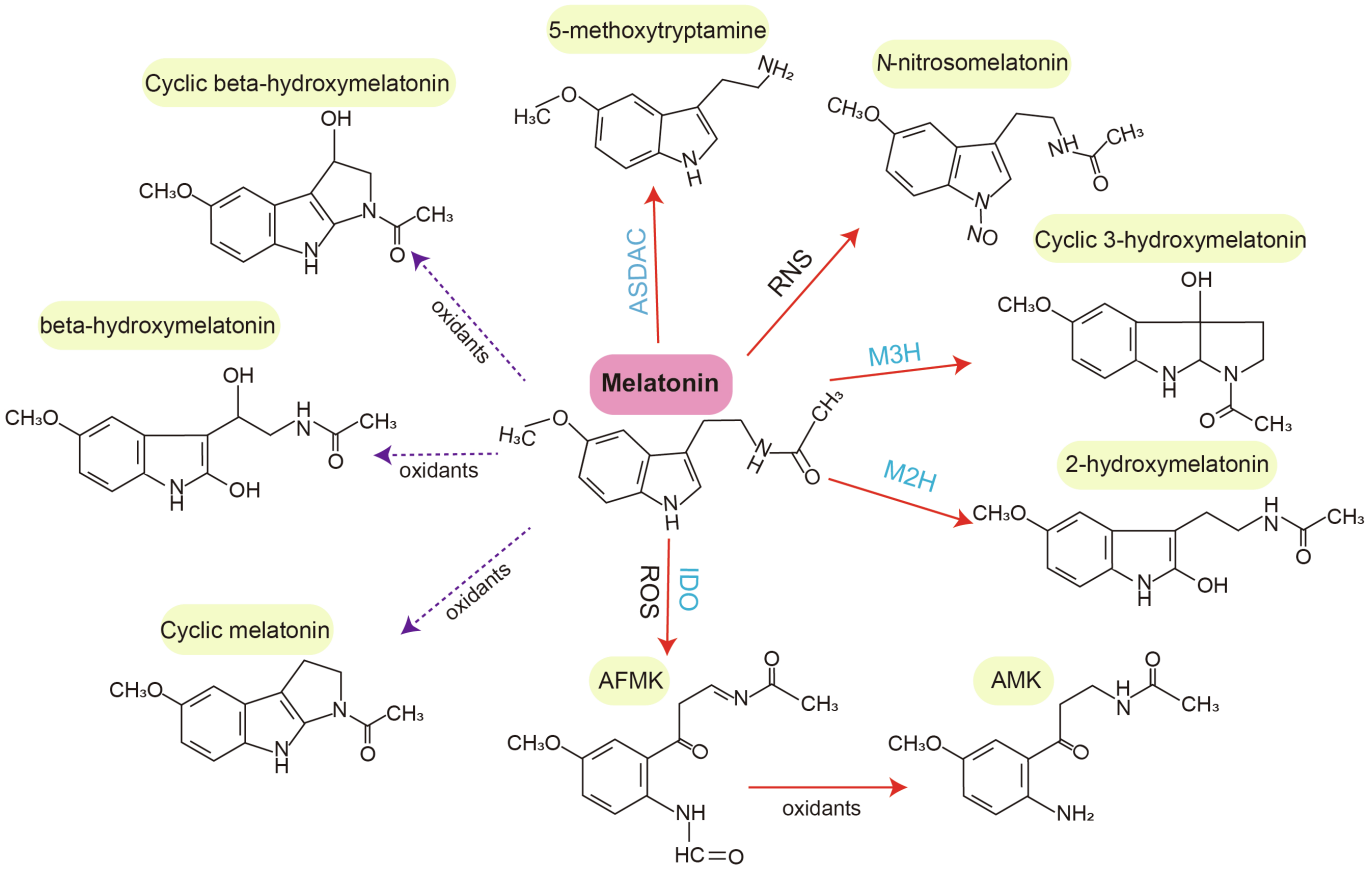
Degradation (catabolism) of melatonin in plants. Melatonin is typically degraded into various metabolites through enzymatic (M3H, M2H, ASDAC, and IDO) and non-enzymatic (oxidants, ROS, and RNS) transformation routes. Red solid arrows indicate confirmed melatonin metabolites. Purple broken arrows indicate potential melatonin metabolites. AFMK, N1-acetyl-N2-formyl-5-methoxykynuramine; AMK, N-acetyl-5-methoxykynuramine; IDO, indoleamine 2,3-dioxygenase; M2H, melatonin 2-hydroxylase; M3H, melatonin 3-hydroxylase; and ASDAC, N-acetylserotonin deacetylase.

The gene responsible for producing c3-OHM, another important melatonin hydroxylation metabolite, has been cloned in rice (Lee et al., 2016). c3-OHM production is catalyzed by M3H, which belongs to the 2-ODD superfamily. It is located in the cytoplasm. Of note, c3-OHM levels exhibit a diurnal rhythm in rice (Choi and Back, 2019). Additionally, M2H and M3H are endemic to terrestrial plants, suggesting that the functions of their enzymatic products are specific to land plants (Lee and Back, 2019). Generally, c3-OHM and 2-OHM are the predominant hydroxylated forms of melatonin found in plants, in contrast to the 6-OHM mainly found in animals. Moreover, 5-methoxytryptamine is another bioactive metabolite. It is formed from melatonin by N-acetylserotonin deacetylase (ASDAC) and from serotonin by ASMT in rice seedlings (Back, 2021). For non-enzymatic melatonin degradation, melatonin can be nitrosated at the nitrogen atom of its indole ring, resulting in the formation of N-nitrosomelatonin (Blanchard et al., 2000; Mukherjee, 2019). The melatonin metabolites beta-hydroxymelatonin, cyclic beta-hydroxymelatonin, and cyclic melatonin are also present in corn and cucumber. They may be produced because of interactions between melatonin and oxidizing agents such as ROS and RNS (Kołodziejczyk et al., 2015; Manchester et al., 2015; Figure 2).

Melatonin coexists with its major metabolites (3-OHM, 2-OHM, and 5-methoxytryptamine) in plant cells (Lee and Back, 2019). Both endogenous melatonin and its metabolisms may be affected by exogenous melatonin (Back, 2021). After exogenous melatonin application in plants, the resulting physiological and biochemical changes and stress responses may differ depending on the changed level of melatonin metabolites and endogenous melatonin (Lee and Back, 2016a). Endogenous levels of melatonin metabolites are generally high in plants, where they exhibit high antioxidant activity.

Many melatonin-mediated abiotic stress responses (e.g., to drought, salt, and heat) and physiological process (e.g., flowering, senescence, somatic embryogenesis, sugar metabolism and secondary metabolism) are related to the mitogen-activated protein kinase (MAPK) cascade pathway, melatonin metabolism (Erland et al., 2018; Kobylińska et al., 2018; Qi et al., 2018; Huang et al., 2019; Liu et al., 2019; Zhang H. et al., 2019; Back, 2021), and other more complicated signaling pathways (hormones, ROS, and Ca^2+^; Arnao and Hernández-Ruiz, 2018; Zhang Y. et al., 2021). Melatonin metabolites can reduce oxidative damage by balancing the redox state (Galano and Reiter, 2018; Yu et al., 2018). However, the signaling pathways of melatonin metabolites in plants have not been fully elucidated.

## Melatonin Functions as an Antioxidant in Plants

### Melatonin as a ROS and RNS Scavenger

Reactive oxygen species are byproducts in plant aerobic metabolic processes (Apel and Hirt, 2004). ROS consist of radical and non-radical oxygen species. They may be as simple as molecules of superoxide or more complex molecules, like hydrogen peroxide (H_2_O_2_) and hydroxyl radical (OḤ; Ray et al., 2012). In plants, H_2_O_2_, O_2_^•−^, OḤ, and ^1^O_2_ are the most common ROS. They are mainly generated in chloroplasts, mitochondria, and peroxisomes under stresses. In chloroplasts, ROS generation depends on the interaction of chlorophyll (chl) and light (Hasanuzzaman et al., 2020). The light energy is captured in photosystem II (PSII) to cause photosynthetic electron transfer. During light-driven photosynthetic electron transport, highly reactive singlet oxygen (^1^O_2_) is formed by transferring the absorbed energy to ground-state oxygen of O_2_ (Li and Kim, 2021). ^3^Chl and ^3^P680 are involved in this process. In addition, O_2_^•−^ is produced in photosystem I (PSI) by Mehler reaction. O_2_^•−^ can be transferred to H_2_O_2_ and O_2_ by SOD, thus generating OḤ *via* the interaction of H_2_O_2_ with transition metal ions (Fe^2+^ and Cu^+^; Hasanuzzaman et al., 2020; Li and Kim, 2021). In mitochondria, Complex I and Complex III are two components of the mitochondrial electron transport chain (mtETC) to covert O_2_ to O_2_^•−^. Then, Mn-SOD and Cu-Zn-SOD catalyze O_2_^•−^ to H_2_O_2_ (Huang et al., 2016; Hasanuzzaman et al., 2020). In addition, oxidative metabolism also exits in peroxisomes. A number of enzymes are implicated in active metabolism of ROS in peroxisomes such as glycolate oxidase, xanthine oxidase and NADPH oxidase (Del Río and López-Huertas, 2016; Kerchev et al., 2016). Peroxisome is also one of the main cellular sites of NO production that exerts a regulatory function of ROS metabolism.

Damage caused by environmental stress in plants typically occurs because of oxidant stress, which manifests as a loss of equilibrium between oxidant and antioxidant chemical species in the cell, thus altering redox homeostasis and accelerating ROS accumulation (Nguyen et al., 2018). ROS can cause oxidative modification of proteins in chloroplasts under stress conditions. For example, excess ^1^O_2_ is reported to oxidize PSII core proteins, polyunsaturated fatty acids (PUFAs) and some specific amino acid residues like tryptophan (Trp), tyrosine (Tyr), phenylalanine (Phe), histidine (His), methionine (Met), and cysteine (Cys; Li and Kim, 2021). O_2_^•−^ and OḤ can oxidize tyrosine (Y) 246 in the D1 protein (Kumar et al., 2021). α-tocopherol plays a significant role in preventing oxidative modification of the D1-Y246 residue (Li and Kim, 2021). ^1^O_2_ can also oxidize a disulfide bond (DSB), thus forming a disulfide crosslink with thiol-containing proteins (Jiang et al., 2021; Li and Kim, 2021). In addition, ROS are utilized as signaling agents by plants and trigger a series of responses that protect the plant against stress factors (Nabi et al., 2019). Among ROS signaling pathways, operational retrograde signaling (ORS) pathways are generally associated with stress. ORS pathways can be mediated by β-cyclocitra (βCC), 3′ (2′)-phosphoadenosine-5′-phosphate (PAP), methylerythritol cyclodiphosphate (MEcPP), and EXECUTER1 (EX1; Kim, 2020; Li and Kim, 2021). Moreover, various hormones (JA, SA, ABA) participate in the crosstalk with ROS to active ROS detoxification under environmental stresses (Lemos et al., 2016; Lv et al., 2019; Postiglione and Muday, 2020; Wang Y. et al., 2021). In addition to hormones, trehalose and anthocyanins are also reported to regulate ROS metabolism (Naing and Kim, 2021; Yang et al., 2022).

NO is an important molecular signal and regulates ROS metabolism (Corpas et al., 2019). Interactions between nitric oxide (NO) and ROS induce radical and non-radical RNS generation under unfavorable conditions. This process is known as nitrosative stress (Corpas and Barroso, 2013; Saddhe et al., 2019). RNS include nitroxyl anion (NO^−^), nitrosonium cation (NO^+^), higher oxides of nitrogen (NO, NO_2_ and higher valence), S-nitrosothiols, and dinitrosyl iron complexes (DNICs; Martínez and Andriantsitohaina, 2009). RNS modify protein activity *via* nitrosylation and interactions between NO and other biomolecules (ferrous heme and catalase; Arora et al., 2016). Moreover, peroxynitrite (ONOO-) and S-nitrosoglutathione (GSNO) derived from NO can modulate the ROS metabolism of peroxisomes through protein posttranslational modifications (Corpas et al., 2019). RNS can also interact with various signaling cascades, such as Ca^2+^-dependent and phytohormone signaling pathways (Arora et al., 2016; Hasanuzzaman et al., 2018). ROS and RNS have dual roles in plant cells. At low levels, they act as intracellular signaling agents and induce a positive response within the antioxidant system. At high levels, they become toxic and damage both cells and proteins. In plants, the genes of receptor kinase, annexin, and peroxisome biogenesis can be induced by ROS (Apel and Hirt, 2004). ROS and RNS can be neutralized or scavenged by a diverse array of enzymatic and non-enzymatic antioxidants (Arnao and Hernández-Ruiz, 2019b). Enzymatic antioxidant agents include superoxide dismutase (SOD), catalase (CAT), ascorbate peroxidase (APX), glutathione (GSH) peroxidase, GSH S-transferase, monodehydroascorbate reductase (MDHAR), peroxidase, and peroxiredoxin. Non-enzymatic compounds include polyphenols, flavonoids, polyamines, ascorbic acid (AsA), GSH, cysteine, alkaloids, proline, carotenoids, tocopherols, and plastoquinone.

Melatonin is one of ROS-regulated molecules (Pardo-Hernández et al., 2020). It is well known for ROS detoxification and an ecologically friendly antioxidant compound. Melatonin neutralizes free radical species *via* single-electron and hydrogen transfer (Galano and Reiter, 2018). It can directly interact with ROS to enhance plant resistance to various stresses by scavenging ROS (O_2_^•−^, OḤ), RNS (nitric oxide and dioxide radicals, azide radicals and peroxynitrite radicals) and other oxidative agents. The most reactive free radical among ROS is the hydroxyl radical (OḤ), which is mainly responsible for the oxidative damage to DNA (Chatgilialoglu et al., 2009; Galano and Alvarez-Idaboy, 2009). Furthermore, melatonin has superior OḤ scavenging activity, compared to mannitol and GSH (Arnao and Hernández-Ruiz, 2019b). Melatonin reduced nickel-induced oxidative damage by decreasing O_2_^•−^ and hydrogen peroxide (H_2_O_2_) production in tomato leaves and roots (Jahan et al., 2020). Melatonin improved high light tolerance of *Arabidopsis* by scavenging H_2_O_2_ and O_2_^•−^ (Yang et al., 2021). Under different abiotic stresses, exogenous melatonin can trigger the biosynthesis of endogenous melatonin to suppress the accumulation of H_2_O_2_, O_2_^•−^ and MDA, thus alleviating cell membrane damage and improving photosynthesis (Chen Y. et al., 2021; Imran et al., 2021). In addition, melatonin derivatives such as c3-OHM and AFMK exhibit high OḤ and hydroperoxyl radical scavenging efficiency (Galano et al., 2014; Galano and Reiter, 2018). Therefore, melatonin and its metabolites act as direct ROS and RNS scavengers in plants under stress.

### Melatonin as a Signaling Molecule

**Figure 3:**
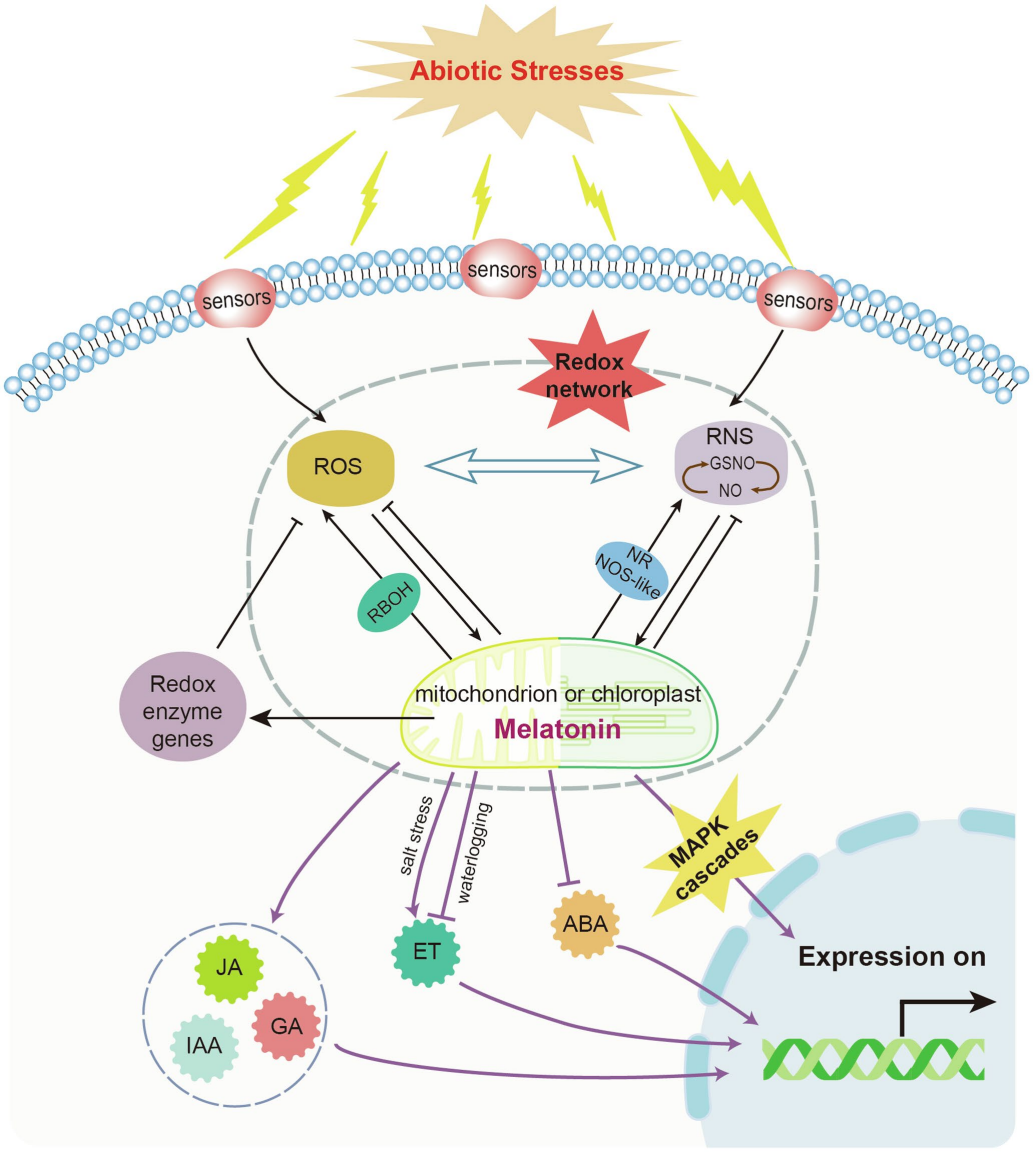
Endogenous melatonin-modulated feedback mechanism in plants under abiotic stresses. Abiotic stresses induce the accumulation of reactive oxygen species (ROS) and reactive nitrogen species (RNS), thus triggering the biosynthesis of endogenous melatonin. RNS and ROS can be scavenged by plant melatonin. Their levels can also be controlled by melatonin-mediated induction of redox enzymes. Mitogen-activated protein kinase (MAPK) cascades and other phytohormone signals are involved in melatonin-modulated transcriptional feedback mechanisms under abiotic stresses. NOS, nitric oxide synthase; GSNO, S-nitrosoglutathione; and RBOH, respiratory burst oxidase homolog.

Melatonin functions as a signaling molecule. Under stress conditions, melatonin can directly enhance antioxidant enzyme activity and indirectly activates the gene expression of stress response systems and antioxidative systems (Figure 3). The expression of antioxidant enzymes (e.g., CAT, POD, and SOD, which detoxify excess H_2_O_2_) is induced by melatonin to restore redox homeostasis and control ROS levels. A number of antioxidant enzyme-related genes are induced by melatonin. For example, melatonin can alleviate oxidative damage caused by salt stress through the enhancement of antioxidant enzyme activity and removal of H_2_O_2_ in cotton (Zhang Y. et al., 2021). However, melatonin induces the expression of respiratory burst oxidase homologs (RBOH) that generate O_2_^•−^, causing an increase in H_2_O_2_ levels (Chen et al., 2017; Arnao and Hernández-Ruiz, 2019a). In plants, melatonin signaling triggers abiotic and biotic stress responses by acting on ROS- and RNS-mediated pathways. It also modulates various antioxidant pathways such as the AsA-GSH cycle (Erland et al., 2017). Melatonin can regulate hydrogen peroxide-mediated signaling pathway to detoxify excess ROS (Imran et al., 2021). In addition, Ca^2+^ signal pathway participates in melatonin-mediated abiotic stress tolerance *via* the regulation of Ca^2+^ signal genes like *CIPK3* and *CIPK9* (Gu et al., 2020). ROS generating enzymes (NADPH oxidase and GOX) are suppressed by the combined application of melatonin and Ca^2+^ (Zhang Y. et al., 2021). Moreover, melatonin regulates stress-related gene expression. For example, melatonin induced the expression of *HSFA2* and heat *HSP90*, which enhance thermotolerance in tomato seedlings (Jahan et al., 2019). In salt stress, ion-response gene expressions (*GhNHX1*, *GhSOS1* and *GhAKT1*) were upregulated by melatonin treatment for better salt tolerance of cotton (Shen et al., 2021). In tomato, carbohydrate metabolism is also demonstrated to be regulated by melatonin treatment (Iqbal et al., 2021). Carbon starvation-induced chlorophyll degradation and leaf senescence were alleviated by exogenous melatonin through upregulating transcript levels of miR171b, thus suppressing the expression of *α-glucan water dikinase* (*GWD*) gene (Wang et al., 2022).

Melatonin can also mediate the NOS-like system to exert physiological functions and benefit plants under stress conditions (Arnao and Hernández-Ruiz, 2018). For example, it interacts with NO to activate the SA-dependent pathway and MAPK cascades in response to biotic stresses (He and He, 2020). In *Arabidopsis*, pathogen attack-induced melatonin biosynthesis can activate MAPK kinase kinases such as oxidative signal-induced kinase 1, followed by the MAPK kinase 4/5/7/9 and MAPK3/6 cascades. This upregulates the expression of several defense genes such as pathogenesis-related protein 1 (*PR1*), isochorismate synthase 1 (*ICS1*), GSH S-transferase 1 (*GST1*), and *APX1* (Lee et al., 2015; Lee and Back, 2016b, 2017). Similar to melatonin, some melatonin metabolites such as 2-OHM and AFMK can activate MAPKs. However, the induction of MAPK pathways by c3-OHM has not yet been reported (Lee and Back, 2016b).

A controversial topic of melatonin research is the regulation of other plant hormones by melatonin. Melatonin regulates the gene expression of multiple enzymes, receptors, and transcription factors that are associated with the biosynthesis and catabolism of IAA, GA, CK, ABA, ET, JA, SA, and BR. These relationships indicate possible crosstalk between melatonin and other phytohormones (Weeda et al., 2014; Arnao and Hernández-Ruiz, 2019a; Figure 3). Melatonin and IAA are structurally related and have the same precursor, tryptophan. Melatonin also upregulates the expression of auxin signaling and efflux genes (*PIN1*, *PIN3*, and *PIN7*), along with adventitious root formation in tomato (Wen et al., 2016). It also influences the biosynthesis and catabolism of ABA in response to various abiotic stresses (e.g., salt, drought, heat, and cold), possibly acting upstream of the ABA pathway (Li et al., 2015; Fu et al., 2017; Li H. et al., 2017; Arnao and Hernández-Ruiz, 2018). In addition, melatonin reprograms the biosynthesis and metabolism of ET and polyamine biosynthesis to improve waterlogging tolerance in alfalfa (Zhang Q. et al., 2019; Gu et al., 2020). Although there is increasing evidence of crosstalk between melatonin and other plant hormones, details regarding the underlying mechanisms are largely unknown.

## Roles of Melatonin in Plant Abiotic Stress Responses

Melatonin has important roles in plant responses to abiotic stress. In this section, recent research concerning melatonin-mediated responses to several major abiotic stress factors will be discussed.

### Drought Stress

Drought restricts plant growth and development. It is also a severe threat to crop productivity and quality because of alterations to the morphological, physiological, biochemical, and molecular features of plants (Cherono et al., 2021). Drought induces oxidative stress and activates stress signaling pathways mediated by ROS, Ca^2+^, and hormones. In addition, drought stress can activate transcription factors such as NACs, MYBs, AP2/EREBPs, bZIPs, HDs, and bHLHs (Zhang J. et al., 2014). Melatonin has an important role in drought response and resistance in plants. Typically, water deficiency promotes melatonin biosynthesis (Shi et al., 2015a). Endogenous melatonin levels change during water deprivation because melatonin biosynthesis genes (e.g., *TDC*, *ASMT*, *COMT*, and *SNAT*) are upregulated. Drought-induced melatonin biosynthesis has been reported in *Arabidopsis*, barley, bermudagrass, apple, grapevine, and rice (Moustafa-Farag et al., 2020).

Exogenous melatonin treatment can trigger an increase in endogenous melatonin levels, thereby improving turgor pressure, cell stability, chlorophyll recovery, and photosynthetic machinery stability under drought stress (Meng et al., 2014; Ding et al., 2018). Furthermore, melatonin treatment can promote seed germination, lateral root formation, hypocotyl elongation, vegetative growth, and fruit quality during drought (Hosseini et al., 2021; Sun et al., 2021). In rapeseed, seed priming with melatonin improves stomatal and ultrastructural traits, as well as osmotic adjustment, thus improving drought tolerance (Khan et al., 2019). Melatonin pretreatment in tomato improves dehydration resistance and promotes vegetative growth by decreasing the compatible solute content (e.g., of proline and soluble sugars) and increasing photosynthetic efficiency (Liu et al., 2015; Ibrahim et al., 2020). In lemon verbena plants, melatonin treatment alleviates the adverse effects of drought, while improving growth and essential oil yields by regulating mineral homeostasis and osmolyte accumulation (Hosseini et al., 2021). Moreover, melatonin-mediated drought tolerance in soybean is associated with glucose metabolism.

Melatonin treatment can also alleviate drought-induced oxidative damage in plant species such as apple, tomato, alfalfa, and wheat (Li et al., 2015; Antoniou et al., 2017; Cui et al., 2017). During oxidative stress, antioxidative enzymes such as APX, CAT, SOD, peroxide dismutase, peroxidase, dehydroascorbate reductase (DHAR) and GSH reductase are activated by melatonin, which suppresses ROS accumulation in drought environments (Alharby and Fahad, 2020; Sadak and Bakry, 2020). In *Medicago sativa*, melatonin serves as a priming agent by modulating nitro-oxidative and osmoprotective homeostasis to resist drought stress (Sun et al., 2021). After melatonin treatment, the AsA-GSH cycle, an integral antioxidant system, is enhanced through the upregulation of related genes (*APX*, *MDHAR*, and *DHAR*) to improve drought stress tolerance (Cui et al., 2017; Tiwari et al., 2021). In addition, MAPKs and transcription factors that improve drought tolerance are regulated by melatonin (Tiwari et al., 2021). In oat seedlings, the expression levels of MAPKs (e.g., *Asmap1* and *Aspk11*) and transcription factor genes (e.g., *WRKY1*, *DREB2*, and *MYB*) are upregulated by melatonin treatment (Gao et al., 2018).

Crosstalk between melatonin and other phytohormones is another crucial drought stress regulatory mechanism. The application of melatonin may alleviate drought-induced suppression of flowering in plants by inducing GA biosynthesis (Tiwari et al., 2020). Drought tolerance is enhanced by interactions between melatonin and ABA, which regulate stomatal closure (Chen et al., 2020; Qi et al., 2021). Melatonin downregulates ABA biosynthesis genes and upregulates ABA catabolism genes (*MdCYP707A1* and *MdCYP707A2*) to maintain stomatal function during drought stress (Li et al., 2015).

### Waterlogging Stress

Waterlogging can affect crop survival, growth, and productivity. This phenomenon severely limits gas diffusion, which results in hypoxic stress caused by anaerobic respiration in the roots, thereby promoting ROS accumulation (Zhang Q. et al., 2019; Gu et al., 2020; Wu Q. et al., 2021). This burst of excessive ROS production causes roots to rot and leaves to wilt (Hossain et al., 2009). Melatonin participates in the regulation of plant responses to waterlogging. When plants are faced with waterlogging stress, their endogenous melatonin levels increase because melatonin biosynthesis genes (e.g., *TDC*, *T5H*, *ASMT*, *COMT*, and *SNAT*) are upregulated (Moustafa-Farag et al., 2020).

Endogenous melatonin levels are positively correlated with waterlogging resistance. Treatment with exogenous melatonin markedly enhances seedling vitality in peach, apple, and alfalfa by regulating endogenous melatonin levels under waterlogging conditions (Zheng et al., 2017; Zhang Q. et al., 2019; Gu et al., 2020). Additionally, melatonin treatment abates stomatal closure, chlorophyll and photosynthesis reduction, and leaf senescence (Zhang Q. et al., 2019; Gu et al., 2020). In *Malus baccata*, exogenous melatonin application induces the post-transcriptional regulation of endogenous melatonin levels during waterlogging (Zheng et al., 2017). In addition, waterlogging-induced oxidative damage is alleviated by melatonin treatment. Melatonin application enhanced the activities of several antioxidant enzymes and reduced H_2_O_2_ concentrations in both leaves and roots of peach seedlings to maintain redox homeostasis under waterlogging stress (Gu et al., 2020).

Additionally, melatonin plays a beneficial role in the regulation of waterlogging-induced hypoxia stress by optimizing metabolism in roots and regulating hypoxia-related genes (e.g., ET response factors and calcineurin B-like protein-interacting protein kinase) in peach seedlings (Gu et al., 2020). ET is an important signal associated with hypoxia stress. ET synthesis and signaling is affected by melatonin, which inhibits the expression of 1-aminocyclopropane-1-carboxylate synthase, 1-aminocyclopropane-1-carboxylic acid oxidase, and ET response factor (Zhang Q. et al., 2019; Gu et al., 2020). Notably, waterlogging induced-ET production is suppressed by treatment with moderate amounts of melatonin, whereas ET biosynthesis is induced by treatment with high concentrations of melatonin (Zheng et al., 2017). Therefore, the crosstalk between melatonin and ET has an important regulatory role in waterlogging stress tolerance.

### Salt Stress

Salt stress has become a severe global problem, limiting agricultural production and leading to substantial economic losses worldwide (Su et al., 2021). Salt has two detrimental effects on plants: osmotic stress and ion poisoning (Van Zelm et al., 2020). Salt stress alters gene expression, mRNA stability, and translational regulation, thus altering the abundances of proteins in plants (Sharma and Dietz, 2009; Van Zelm et al., 2020). Plant phospholipid signals, protein kinase signals, and ABA are involved in responses to salt stress.

Salt tolerance is enhanced by melatonin in various plants such as maize, wheat, cucumber, tomato, cotton, and rice (Liang et al., 2015; Zhou et al., 2016; Chen et al., 2018; Ke et al., 2018; Zhang Y. et al., 2021). Salt stress can affect the expression patterns of key melatonin biosynthetic enzymes and increase endogenous melatonin content (Arnao and Hernández-Ruiz, 2009). Moreover, the overexpression of *SNAT* can significantly improve plant salt tolerance. For example, *VvSNAT1* overexpression *Arabidopsis* lines exhibit greener leaves, enhanced growth, and higher germination rates under salt stress, compared with wild-type lines (Wu Y. et al., 2021). In contrast, SNAT inhibition lowers endogenous melatonin levels, thereby increasing the sensitivity of rice to salt stress (Byeon and Back, 2016).

Exogenous melatonin treatment helps to protect plants against salt stress by regulating antioxidant enzyme expression and activity, polyamine metabolism, and NO signaling (Zhan et al., 2019). The activation of major antioxidant enzymes decreases salinity-induced ROS and H_2_O_2_ levels. In addition, crosstalk between melatonin and NO regulates redox equilibrium *via* the differential expression of *copper/zinc-SOD* and *manganese-SOD*, and by modulating both GSH levels and GSH reductase activity in cotyledons under salt stress (Arora and Bhatla, 2017). Moreover, melatonin induces crosstalk between NO and hydrogen sulfide, thus improving salt stress tolerance in pepper (Kaya et al., 2020). Molecular hydrogen triggers melatonin signaling by activating SNAT-dependent melatonin production in *Arabidopsis* (Su et al., 2021).

Ion homeostasis is vital for plant survival under salt stress. There is evidence that melatonin assists with ion homeostasis maintenance, particularly concerning K^+^ and Na^+^. Melatonin-mediated ion homeostasis is associated with the expression of *NHX* and *AKT*, which encode two important ion channels (Li et al., 2012). The NHX family is related to K^+^ and Na^+^ antiporters. Na^+^ accumulates in vacuoles when *NHX* is upregulated (Munns and Tester, 2008). Under salt stress, the salt overly sensitive signaling pathway is triggered to transport Na^+^ out of the cells, or to activate an unknown transporter that confines Na^+^ to the vacuole.

Exogenous melatonin treatment leads to triacylglycerol breakdown, fatty acid β-oxidation, and energy turnover to maintain the activity of the plasma membrane H^+^-ATPase. These processes mediate K^+^/Na^+^ homeostasis and provide evidence for the mediation of seed germination by melatonin in cucumber under salt stress (Zhang N. et al., 2017). In addition, Na^+^ and Cl^−^ accumulation is inhibited in rice roots and leaves after exogenous melatonin application by enhancing the transcription of *OsSOS1* in roots and of *OsCLC1* and *OsCLC2* in roots and leaves (Li X. et al., 2017).

ET biosynthesis is promoted by exogenous melatonin treatment during salt stress in grapevine. Endogenous ET enhancement is regulated by *MYB108A* expression, which activates the expression of the ET biosynthesis-related gene *ACS1* (Xu et al., 2019). GA and ABA, as well as antioxidant enzymes, are involved in melatonin-mediated seed germination under high salt conditions in cucumber (Zhang H. et al., 2014). Melatonin coordinates with BR during salt stress by regulating genes involved in BR biosynthesis and signal transduction in cotton. In addition, the Ca^2+^ signal transduction pathway has a pivotal role in melatonin-mediated salt tolerance (Zhang Y. et al., 2021).

### Cold (Chilling) Stress

Cold stress exerts destructive effects on crop growth and productivity. It can lead to redox imbalance and induce excessive ROS accumulation. In response to cold stress, plants increase ABA synthesis, thus activating the C-repeat binding factor pathway (Knight et al., 2004). Extreme cold environments typically induce melatonin accumulation to protect plants against fatal injuries. Increasing endogenous melatonin levels can improve plant cold tolerance. For example, *SNAT* transgenic rice are less sensitive to cold than wild-type plants, indicating the protective role of melatonin in cold resistance (Kang et al., 2010). There is evidence that melatonin functions as a long-distance signal that can be transported from roots to shoots (Tan et al., 2007). In grafted watermelons, melatonin improves cold tolerance *via* root-to-shoot communication and transportation (Li H. et al., 2021).

Exogenous melatonin treatment can maintain the quality of fruits, vegetables, and cut flowers by conferring chilling tolerance under cold storage. For example, pre-storage treatment of loquat fruit with melatonin triggers the accumulation of phenolic compounds and a reduction in lignin, thereby alleviating flavor and nutrition loss induced by chilling injury under cold storage (Wang D. et al., 2021). Melatonin treatment in Bermuda grass can improve the levels of secondary metabolites including amino acids, organic acids, carbohydrates, and sugars, thereby enhancing cold tolerance (Hu et al., 2016). The application of 2-hydroxymelatonin, a metabolite of melatonin, may also improve cold and drought tolerances in tobacco, tomato, and cucumber (Lee and Back, 2019).

Similar to its effects in the context of other stress factors, exogenous melatonin application can alleviate cold damage through the activation of antioxidant enzymes, as well as reductions of both ROS accumulation and lipid peroxidation (Hu et al., 2016). For example, cut flowers treated with melatonin had lower H_2_O_2_ concentrations and increased 2,2-diphenyl-1-picrylhydrazyl (DPPH) scavenging capacity under cold stress than did untreated flowers (Aghdam et al., 2019). Chilling tolerance in tomato increases because of arginine-dependent NO accumulation after melatonin treatment. Under cold stress, melatonin upregulates genes related to stress responsive pathways such as C-repeat binding factor/dehydration-responsive element-binding protein (CBF/DREB), *cold regulated 15a* (*COR15a*), *calmodulin binding transcription activator 1* (*CAMTA1*), and zinc finger transcription factors 10 and 12 (ZAT 10 and ZAT 12; Zhao et al., 2017; Guo et al., 2018; Wang et al., 2018). The rhythmic expression patterns of circadian clock genes are also altered by melatonin supplementation in hulless barley seedlings, regulating their growth and enhancing cold resistance (Chang et al., 2021).

Additionally, the expression patterns of phytohormone biosynthesis genes are modulated by exogenous melatonin. For example, the expression levels of a key gene (*ClAOC1*) in JA biosynthesis, and a key gene (*ClAMI1*) in IAA biosynthesis are upregulated in watermelon leaves after melatonin application under cold stress (Chang et al., 2020; Li H. et al., 2021). Consequently, the JA and IAA contents increase, thus improving photosynthesis and redox homeostasis (Chang et al., 2021).

### Heat Stress

Heat stress is becoming a global concern because of global warming. Frequent heat waves have a profound impact on terrestrial plants. Heat stress affects plants at the physiological (e.g., photosynthesis, cell membrane thermostability, and oxidative damage), transcriptional, post-transcriptional (e.g., non-coding RNAs), and epigenetic (e.g., DNA methylation, histone modification, and chromatin remodeling) levels (Zhao et al., 2020). During heat stress, endogenous melatonin levels increase, thereby improving plant thermotolerance. In tomato, *COMT1* and *TDC* silencing aggravates high temperature-induced oxidative damage by suppressing endogenous melatonin biosynthesis (Ahammed et al., 2019). Furthermore, *ASMT* and *SNAT* overexpression significantly improves thermotolerance by increasing endogenous melatonin (Xu et al., 2016; Wang et al., 2020).

Under heat stress, exogenous melatonin treatment can enhance plant antioxidant defense systems *via* control of ROS accumulation and enhancement of proline metabolism. In tomato seedlings, melatonin treatment promotes thermotolerance by balancing redox homeostasis, while modulating polyamine and nitric oxide biosynthesis (Jahan et al., 2019). In wheat, photosynthetic potential is maintained by interactions between melatonin and hydrogen sulfide under heat stress (Iqbal et al., 2021). Upon exposure to high temperatures, exogenous melatonin treatment can alleviate heat damage in plants by mediating the activity of HSFs, which are the master mediators of heat responses. In *Arabidopsis*, melatonin-mediated thermotolerance was associated with the upregulation of *HSFA2* and *HSA32*, as well as *HSP90* and *HSP101* (Shi et al., 2015b). In tomato, melatonin treatment upregulated *HSP* gene expression and increases HSP production, which then repaired denatured or damaged proteins after heat exposure (Xu et al., 2016). Furthermore, *HSP40* interacted with *SlSNAT* to regulate melatonin biosynthesis and promote thermotolerance by maintaining Rubisco enzyme stability under heat stress (Wang et al., 2020). In addition, heat-induced leaf senescence is suppressed by melatonin treatment, which regulates ABA and CK biosynthesis and signaling pathways (Zhang J. et al., 2017). Exogenous melatonin reduces heat-induced damage by upregulating SA production and downregulating ABA levels in soybean seedlings (Imran et al., 2021).

### Heavy Metal Toxicity

Heavy metals are toxic to plants. Heavy metal stress can repress root growth and promote the leaf senescence by inducing ROS accumulation and damaging chloroplasts. In addition, Calvin cycle enzymes, photosynthesis, and carbohydrate metabolism are also inhibited by heavy mental toxicity (Hasan et al., 2015, 2019; Ni et al., 2018). Toxicity caused by lead (Pb), copper, vanadium, and aluminum can disrupt redox homeostasis and accelerate the accumulation of redox agents (Nagajyoti et al., 2010).

Melatonin treatment can alleviate heavy metal-induced suppression of root growth and activity (Ni et al., 2018; Jahan et al., 2020). Endogenous melatonin production can be induced by treatment with exogenous melatonin to strengthen heavy metal tolerance. In wheat seedlings, melatonin treatment promoted endogenous melatonin biosynthesis in shoots and roots. The enhancement of endogenous melatonin alleviated cadmium (Cd) toxicity by balancing H_2_O_2_ homeostasis and activating antioxidant enzymes (Ni et al., 2018). Exogenous melatonin treatment also enhances the antioxidant potential of plants and reduces lipid peroxidation under aluminum stress (Sun et al., 2020). In tomato, melatonin effectively ameliorated Cd-induced toxicity by enhancing H^+^-ATPase activity, increasing GSH and phytochelatin contents, and facilitating Cd sequestration in plant cells (Hasan et al., 2015). In addition, melatonin treatment alleviates nickel phytotoxicity by improving gas exchange, while increasing the contents of photosynthetic pigments, minerals, nutrients, and secondary metabolites (Jahan et al., 2020). NO signaling is involved in melatonin-induced antioxidant defenses. NO has a vital role in re-establishing redox homeostasis in plants under heavy metal stress. Crosstalk between melatonin and NO improves Pb and Cd stress tolerance (Kaya et al., 2019; Okant and Kaya, 2019).

In addition to regulating antioxidant levels, melatonin mediates heavy metal tolerance by enhancing heavy metal uptake and sequestration. Cd, vanadium, and copper are excluded from or sequestered in plant cells after exogenous melatonin treatment (Nawaz et al., 2018; Cao et al., 2019; Hasan et al., 2019). Notably, melatonin treatment balances endogenous GA and ABA levels and improves thiol-mediated detoxification in two indica rice. Furthermore, melatonin reduces Pb damage in radish plants *via* DNA demethylation of metal transporters and antioxidant genes (Tang et al., 2021).

### Light Stress

Light is dispensable to plants. However, it is also an environmental stressor that causes photodamage and overaccumulation of ROS in plants. Ultraviolet (UV) light can induce the generation of free radicals in plants (Demarsy et al., 2018; Nazir et al., 2020). Exposure to high UV radiation for a long time can result in damage of the nuclear membrane and generally cause changes in the ultrastructure of various cellular components in many plant species.

One important regulator of melatonin biosynthesis is light (Tan et al., 2007; Zhang H. et al., 2019). In plants, it was observed that the concentration of endogenous melatonin was increased after UV-B treatment for short time, suggesting that endogenous melatonin is involved in UV-B response (Afreen et al., 2006; Wei et al., 2019). Exogenous melatonin increased the content isoflavone monomers in 4-day-old germinated soybeans under UV-B stress (Yin et al., 2022). In *Arabidopsis*, melatonin also participated in UV-B signaling pathway by delaying and subsequently enhancing expression of *COP1*, *HY5*, *HYH*, and *RUP1/2* to mediate UV-B stress tolerance (Yao et al., 2021). Under high light stress, melatonin can improve photosynthesis, alleviate inhibitory effect of stomatal apertures and relieve the oxidative damage to cells by regulating redox state (Wei et al., 2019; Haskirli et al., 2021; Yang et al., 2021). In contrast, snat1 knockout mutants of *Arabidopsis* failed to induce melatonin biosynthesis and lower expression of ROS-responsive genes, resulting in sensitivity to high light stress (Lee and Back, 2018). In addition, melatonin may regulate flavonoids to mediate light tolerance (Wei et al., 2019; Nazir et al., 2020). Flavonoids are also important antioxidants (Shen et al., 2022), however, the interaction of melatonin and flavonoids need further investigation.

### Other Abiotic Stresses

Melatonin functions on other abiotic stress factors have been discussed. Potassium deficiency tolerance is a vital problem to crop production. Melatonin can enhance potassium content in *Malus* under different stress conditions and promote potassium deficiency tolerance in wheat (Li et al., 2016; Li G. et al., 2021). *TaHAK1* regulated by *TaNAC71* played an important role in MT-mediated potassium deficiency tolerance in wheat (Li G. et al., 2021). In addition, plastic pollution has received a great concern worldwide. Melatonin was reported to reduce the nanoplastic uptake by roots and their translocation to shoots by regulating the expression of genes associated with aquaporin (*TIP2-9*, *PIP2*, *PIP3*, *PIP1-5* and *PIP1.2*). Melatonin enhanced the tolerance to nanoplastic toxicity by maintaining a better redox homeostasis and ameliorating the negative effects of nanoplastics on carbohydrate metabolism (Li S. et al., 2021).

## Exogenous Melatonin Application

Exogenous melatonin treatment can improve the tolerance of plants to various abiotic stresses (e.g., salt, drought, waterlogging, heat, cold, and heavy metal toxicity) in several plant species. These improvements in stress tolerance are largely associated with the induction of endogenous melatonin production after exogenous melatonin treatment. In addition, these improvements are typically dose-dependent. Treatment with low melatonin concentrations typically induces stronger stress tolerance responses than does treatment with high melatonin concentrations (Zhang et al., 2015).

Overall, melatonin has great potential for use as an anti-stress agent in agricultural crop production applications. Recent reports of exogenous melatonin treatment for abiotic stresses are summarized in Table 1.

**Table 1:** Exogenous melatonin application to plants under abiotic stresses.

Species	Stress level	Melatonin level	Main effects	References
**Drought stress**				
*Glycine max*	Drought stress (45% soil water), 72 h	100 μΜ	Promote sugar metabolism and production and repair the membrane damage by regulating gene expression and enzyme activities (sucrose phosphate synthase, sucrose synthase, sucrose invertase, β-amylase) of sugar metabolism	Cao et al., 2021
*Zea mays*	Drought stress (40%–45% soil water), 7 days	100 μΜ	Improve resistance to drought stress by enhancing the activity of SOD, CAT, POD, APX, enhancing the levels of osmo-protectants and controlling root growth	Ahmad et al., 2021
*Zea mays*	Drought stress (50% soil water), 6 days	100 μΜ	Enhance leaf gas exchange, regulate carbon and nitrogen metabolism *via* promoting the activity of SPS, AGPase, PEPC, CS, NR, NiR, GS, and GOGAT	Alharby and Fahad, 2020
*Zea mays*	Withholding water, 8 days	1 mΜ	Exert a protective effect on drought tolerance by regulating ABA and JA, improving photosystem II efficiency and photochemistry	Fleta-Soriano et al., 2017
*Gossypium hirsutum*	Drought stress (45 ± 5% soil water), 9 days	200 μΜ	Improve antioxidative capacity by promoting the expression of *Cu/ZnSOD*, *CAT*, *POD* and increasing AsA content and the activity of APX, GR and DHAR	Hu et al., 2021
*Lippia citriodora*	Drought stress (50% and 25% soil water), 45 days	100 μΜ	Minimize drought effects by enhancing antioxidative capacity, keeping mineral balance and regulating ABA content	Hosseini et al., 2021
*Medicago sativa*	Withholding water, 7 days	10 μΜ	Improve nitro-oxidative homeostasis through regulating RNS metabolic enzymes, limit cellular redox disruption through the regulation of the mRNA levels of antioxidant and redox-related components	Antoniou et al., 2017
**Waterlogging stress**				
*Sorghum bicolor*	Place seedlings into pots lacking holes for drainage, 14 days	100 μΜ	Strengthen waterlogging tolerance by increasing soluble protein contents and improving the activity of POD, CAT, APX	Zhang R. et al., 2021
*Prunus persica*	Keep water levels at 2 cm above the soil surface, 12 days	200 μΜ	Improve antioxidant enzyme activities, control anaerobic respiration in roots through enhancing aerenchym, regulating the Ca^2+^ signal genes (*CIPK3* and *CIPK9*), metabolism-related genes (*ADH6*, *PDC2*, and *LDHa*) and ethylene synthesis genes (*ACS* and *ACO*)	Gu et al., 2020
*Medicago sativa*	Keep water levels at 1 cm above the soil surface, 10 days	100 μΜ	Alleviate growth inhibition and membrane damage by enhancing polyamines (Put, Spd and Spm) content, decreasing MDA content regulating the expression of key genes involved in polyamine metabolism (*SAMDC*, *SPDS*, *SPMS*, *ADC*, *DAO* and *PAO*) and decreasing ethylene levels	Zhang Q. et al., 2019
**Salt stress**				
*Oryza sativa*	150 mM NaCl solution treatment, 7 days	200 μΜ	Reduce membrane damage and improve antioxidant capacity by enhancing antioxidant enzyme activity (SOD, CAT, POD, APX, GR) and antioxidant content (proline, glycine betaine, flavonoid, total phenol)	Yan et al., 2021
*Oryza sativa*	Two salt treatments (150 and 200 mM NaCl), 7 days	75 μΜ	Strengthen root vigor by enhancing antioxidant enzyme activity, reducing contents of Na^+^ and Cl^−^ and increasing transcription of *OsSOS1* in roots	Li X. et al., 2017
*Gossypium hirsutum*	100 mM NaCl treatment, 8 days	1 μΜ	Enhance photosynthetic capacity and osmotic regulation by enhancing antioxidant system and ion-response gene expression (*GhNHX1*, *GhSOS1* and *GhAKT1*)	Shen et al., 2021
*Gossypium hirsutum*	100 mM NaCl treatment, 12 h	20 μM	Strengthen salt tolerance by scavenging ROS and Ca^+^ signal transduction	Zhang Y. et al., 2021
*Brassica juncea*	150 mM NaCl treatment, 16 days	1 μΜ	Recover salinity-damaged plants by improving amino acid, protein, and SA contents; decreasing ABA content	Park et al., 2021
*Zea mays*	100 mM NaCl treatment, 8 days	1 μΜ	Achieve salt tolerance by regulating the content of sucrose, fructose and proline, suppressing the accumulation of H_2_O_2_ and MDA for better osmotic adjustment and ion balance	Ren et al., 2020
*Hevea brasiliensis*	1% NaCl treatment, 3 days	100 μΜ	Enhance photosynthesis and salt tolerance by increasing flavonoid content and suppressing H_2_O_2_ accumulation	Yang et al., 2019
*Vitis vinifera*	100 mM NaCl treatment, 3 days	50 μΜ	Improve salt tolerance by increasing ACC content and ethylene biosynthesis mediated by *MYB108A*	Xu et al., 2019
*Brassica napus*	125 mM NaCl treatment, 24 h	1 μΜ	Promote salinity tolerance through reestablished redox and ion homeostasis, modulated antioxidant defense related genes and the regulation of *NHX1* and *SOS2* expression	Zhao et al., 2018
**Heat stress**				
*Triticum aestivum*	40°C for 15 days at 6 h time duration every day	100 μM	Protect heat stress-induced photosynthetic inhibition *via* a crosstalk with H_2_S that regulates carbohydrate metabolism	Iqbal et al., 2021
*Glycine max*	42°C, 7 days	100 μM	Increase total phenolic and flavonoid contents, enhance PA and SA biosynthesis, decrease ABA content *via* down-regulation of the *gmNCED3* and up-regulation of catabolic genes (*CYP707A1* and *CYP707A2*) and promote ROS detoxification *via* the hydrogen peroxide-mediated signaling pathway	Imran et al., 2021
*Solanum lycopersicum*	42°C, 24 h	100 μM	Improve antioxidant activities by regulating their related gene expression and the ascorbate-glutathione cycle, detoxify excess ROS *via* hydrogen peroxide-mediated signaling pathway (induction of *RBOH*, *P5CS*, *HSP90*), increase PA and NO contents	Jahan et al., 2019
*Actinidia deliciosa*	45°C, 8 h	200 μM	Enhance antioxidant enzyme activities (POD, CAT, SOD) and regulate key AsA-GSH cycle enzymes (APX, MDHAR, DHAR, GR)	Liang et al., 2018
*Lolium perenne*	38/33°C day/night, 28 days	20 μM	Increase t-ZR contents by regulating CK biosynthesis (*LpIPT2*, *LpLOG1*) and signaling gene (*LpARR1*, *LpARR10*, *LpARR5*, *LpARR17*), decrease ABA content by regulating biosynthesis (*LpZEP*, *LpNCED1*) and signaling genes (*LpABI3*, *LpABI5*)	Zhang J. et al., 2017
**Cold (chilling) stress**				
*Cucumis sativus*	5°C, 72 h	100 μM	Increase NR activity and NO content, upregulate NR-relative mRNA expression and induce chilling response genes (*ICE1*, *CBF1* and *COR47*)	Feng et al., 2021
*Cucumis sativus*	15/8°C day/night, 8 days	200 μM	Upregulate *CsZat12* expression, modulate the PA (Put, Spd and Spm) metabolism and ABA metabolism *via* the regulation of *CsNCED1*, *CsNCED2*, *CsCYP707A1* and *CsCYP707A2*	Zhao et al., 2017
*Citrullus lanatus*	4°C, 24 h	150 μM	Increase MeJA content	Li H. et al., 2021
*Citrullus lanatus*	4°C, 72 h	1.5 μM	Enhance photosystem II and AsA-GSH cycle; mediate CBF-responsive pathway by upregulating *ClCBF1* expression; increase IAA content and decrease ABA content	Chang et al., 2020
*Capsicum annuum*	4°C ± 0.5°C, 20 days	100 μM	Reduce cell structure damage; increase membrane lipid and proline contents, keep higher unsaturated: saturated fatty acid ratio	Kong et al., 2020
*Lycopersicon esculentum*	4°C ± 0.5°C, 28 days	100 μM	Enhance H-ATPase, Ca-ATPase, cytochrome c oxidase (CCO), and succinate dehydrogenase (SDH) enzyme activity, inhibit the transcription of *CaNAC1*, increase unSFA/SFA ratio	Jannatizadeh et al., 2019
**Heavy mental stress**				
*Oryza sativa*	150 μM sodium arsenate (As), 2 days	20 μM	Decrease arsenic bioaccumulation *via* modulating the expression levels of selected arsenic transporters (*OsNramp1*, *OsPT2*, *OsPT8*, *OsLsi1*) and controlling endogenous phytohormone homeostasis	Samanta et al., 2021
*Raphanus sativus* var*. radculus pers*	200 mg L^−1^ Pb(NO_3_)_2_ treatment	50 μM	DNA demethylation of metal transporters n (e.g., *RsABCF5*, *RsYSL7* and *RsHMT*) and antioxidant genes (e.g., *RsAPX2*, *RsPOD52* and *RsGST*)	Tang et al., 2021
*Triticum aestivum*	30 mM AlCl_3_, 12 h	/	Alleviate Al toxicity by augmenting oxidants, enhance exclusion of Al from root apex by altering cell wall polysaccharides and increasing pectin methylesterase (PME) activity	Sun et al., 2020
*Triticum aestivum*	200 mM for CdCl_2,_ 12 h	50 mM	Balance hydrogen peroxide homeostasis *via* maintaining the hydrogen peroxide homeostasis and the regulation of the antioxidant systems	Ni et al., 2018
*Medicago truncatula*	7.52 g L^−1^ Pb solution, 12 weeks	10 μM	Improve AM symbiosis and induce *MtPT4* expression by inhibiting Pb uptake, make a synergistic effect on Pb stress tolerance	Zhang et al., 2020
*Malus baccata*	30 μM Cd, 20 days	100 μM	Regulate Cd uptake, transport, and detoxification by altering the mRNA levels of several genes (*HA7*, *NRAMP1*, *NRAMP3*, *HMA4*, *PCR2*, *NAS1*, *MT2*, *ABCC1*, and *MHX*)	He et al., 2020
*Solanum lycopersicum*	Nickel chloride (NiCl_2_·6H_2_O), 14 days	100 μM	Enhance photosynthesis, improve secondary metabolism (total phenols, flavonoids and anthocyanins) and alters the detoxification related gene expression (*SOD*, *CAT*, *APX*, *GR*, *GST*, *MDHAR*, and *DHAR*)	Jahan et al., 2020
*Solanum lycopersicum*	100 μM Cd, 15 days	100 μM	Regulate sulfur metabolism by suppressing sulfate transporter *(SUT)1* and *SUT2* genes and decreasing transcript levels of the *ATPS*, *APSR*, *SiR* and *OASTL* genes to enhance Cd detoxification	Hasan et al., 2019
*Carthamus tinctorius*	50 μM Pb(NO_3_)_2_, 14 days	100/150/200/300 μM	Alleviate Pb toxicity by reducing Pb uptake and its root-to-shoot translocation along with stimulating activity of SOD, APX, CAT, GPX and GR	Namdjoyan et al., 2019
*Arabidopsis thaliana*	8% w/v NaClO_3_, 15 min	1 μM	Increase root growth by interfering with NO-mediated reduction of cell division cycle progression	Zhang J. et al., 2019
*Zea mays*	PbCl_2_·2.5 H_2_O, 4 weeks	0.05/0.1 mM	Alleviate Pb toxicity by increasing proline levels, NO levels and antioxidant enzyme activities (SOD, CAT and POD)	Okant and Kaya, 2019
*Nicotiana tabacum*	100 μM Cd (CdCl_2_), 7 days	100 μM	Weaken Cd uptake by regulating genes expression of *IRT1*, *Nramp1*, *HMA2*, *HMA4* and *HMA3*	Wang et al., 2019
*Cucumis sativus*	80 μmol·L^−1^ Cu^2+^ (CuSO_4_), 2 weeks	10 nM	Enhance the cell wall capacity for binding copper by increasing the gene expression of *CesA*, *CSL*, *PME* and *XTH*, maintain nutrient elements homoeostasis, activate carbon metabolism *via* the upregulation of *ADH*, *ALDO*, *ENO*, *GAPDH*, *GPI*, *PDC and PGK*	Cao et al., 2019
*Citrullus lanatus*	50 mg/L Vanadium, 7 days	0.1 μM	Enhanced the activity of SOD and CAT, regulate relative transcript expression of respiratory burst oxidase genes (*Cla000765* and Cla001877) and chlorophyll degradation related genes (*Cla013675*, and *Cla006037*)	Nawaz et al., 2018
**Light stress**				
*Glycine max*	40 μW/cm^2^ UV-B radiation, 2 and 4 days	25 μM	Remove UV-B stress by regulating isoflavone metabolism-related enzyme activities (PAL, C4H, 4CL) and enhancing the content of total flavonoids and isoflavone monomers	Yin et al., 2022
*Arabidopsis thaliana*	0.7 μmol·m^−2^·s^−1^ UV-B, 6 h	100 μM	Mediate UV-B signaling pathway by delaying and enhancing expression of *COP1*, *HY5*, *HYH*, *and RUP1/2*	Yao et al., 2021
*Arabidopsis thaliana*	UV-B (46 and 92 kJ·m^−2^·d^−1^), 90 and 180 min	100 μM	Regulate the expression of *glutathione peroxidase 2* (*GPX2*) and *GPX7*, decrease the expression of *alternative oxidase 1a* (*AOX1a*) and *AOX1d*	Haskirli et al., 2021
*Arabidopsis thaliana*	High light (1,000 μmol·m^−2^ ·s^−1^), 3 h	100 μM	Protected photosystem protein by inhibiting the accumulation of H_2_O_2_ and promoting the activity of SOD, POD, APX and GPX	Yang et al., 2021
*Malus hupehensis*	UV-B dosage of 0.24 W·m^−2^ and UV-B dosage of 0.45 W·m^−2^	1 μM	Improve UV-B tolerance by suppressing H_2_O_2_ accumulation and increasing phenolic compounds (chlorogenic acid, phloridzin and quercetin-3-galactoside)	Wei et al., 2019

## Conclusions and Future Perspectives

**Figure 4:**
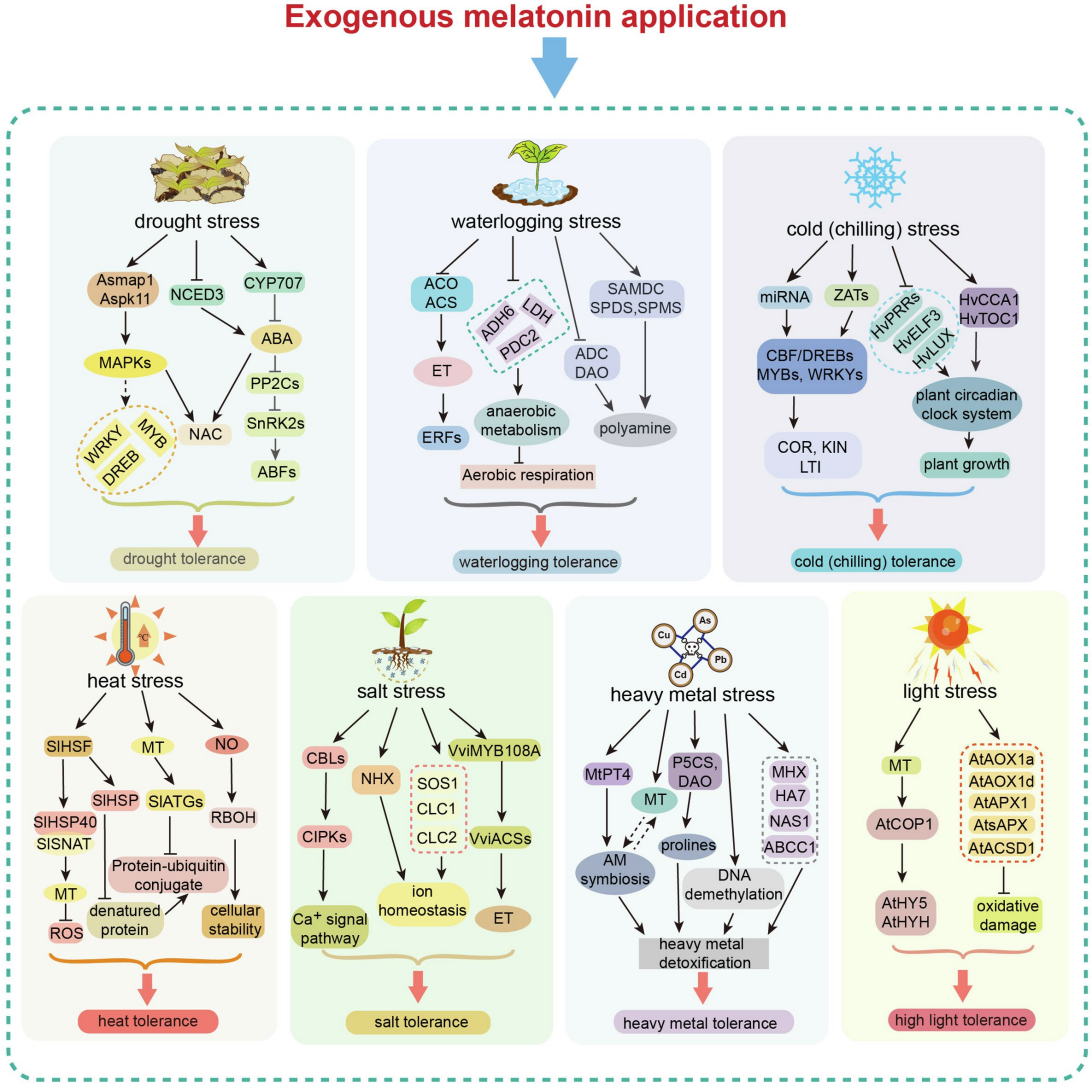
Exogenous melatonin-mediated regulation in response to different abiotic stresses. Exogenous melatonin application induces changes in gene expression in different abiotic stress response pathways, resulting in enhanced tolerance to major abiotic stresses (drought, waterlogging, heat, cold, salt, heavy metal toxicity and light). MAPK, mitogen-activated protein kinase; NCED3, nine-cis-epoxycarotenoid dioxygenase 3; NAC, N-acetylcysteine; DREB, dehydration responsive element binding; CYP, cytochrome P450; PP2C, protein phosphatase 2C; SnRK, sucrose non-fermenting–related related kinase; ABF, abscisic acid responsive element-binding factor; ABA, abscisic acid; ACO, acyl-CoA oxidase; ACS, acetyl-CoA synthetase; ET, ethylene; ERF, ETS2 repressor factor; ADH, alcohol dehydrogenase; LDH, lactate dehydrogenase; PDC2, pyruvate decarboxylase-2; SAMDC, S-adenosylmethionine decarboxylase; SPDS, spermidine synthase; SPMS, spermine synthase; ADC, arginine decarboxylase; DAO, di-amine acid oxidase; CBF/DREB, dehydration responsive element binding/C-repeat binding factor; ZAT, zinc transporter; COR, cold-regulated gene; KIN, Kin17 DNA and RNA binding protein; LTI, low-temperature-induced genes; PRRs, pseudo-response regulators; ELF3, E74 like ETS transcription factor 3; CCA1, circadian colck associated 1; TOC1, timing of CAB expression 1; HSF, heat shock facter; HSP, heat shock protein; SNAT, serotonin N-acetyltransferase; MT, melatonin; RBOH, respiratory burst oxidase homolog; ATG, autophagy-related; CBL, calcineurin B-like proteins; CIPK, CBL-interacting protein kinases; NHX, Na^+^/H^+^ exchanger protein; SOS1, SOS Ras/Rac guanine nucleotide exchange factor 1; CLC, chloride channel; PT4, phosphate transporter 4; AM, arbuscular mycorrhizal; P5CS, delta 1-pyrroline-5-carboxylate synthase; DAO, D-amino acid oxidase; MHX, magnesium/proton exchanger; NAS1, nicotianamine synthase 1; nicotianamine synthase 1; COP1, constitutive photomorphogenic 1; HY5, elongated hypocotyl 5; HYHAPX, hy5 homolog; APX, ascorbate peroxidase; AOX, aldehyde oxidase; and ACSD, 2-amino-3-carboxymuconate-6-semialdehyde decarboxylase.

Plant melatonin is important for alleviating damage caused by various abiotic stresses. In this review, we summarized the regulatory mechanisms that drive melatonin-mediated abiotic stress tolerance in plants. Plant tolerance is typically enhanced by melatonin *via* the following pathways. Melatonin can act as a direct scavenger of ROS and RNS to improve antioxidant capacity. It also acts as a signaling molecule, regulating the expression of genes involved in stress responses, antioxidant production, and phytohormone pathways (e.g., ABA, ET, and JA; Figure 4). Moreover, melatonin affects the redox network. It can interact with NO to regulate redox homeostasis, thus maintaining antioxidant potential during abiotic stress.

**Figure 5:**
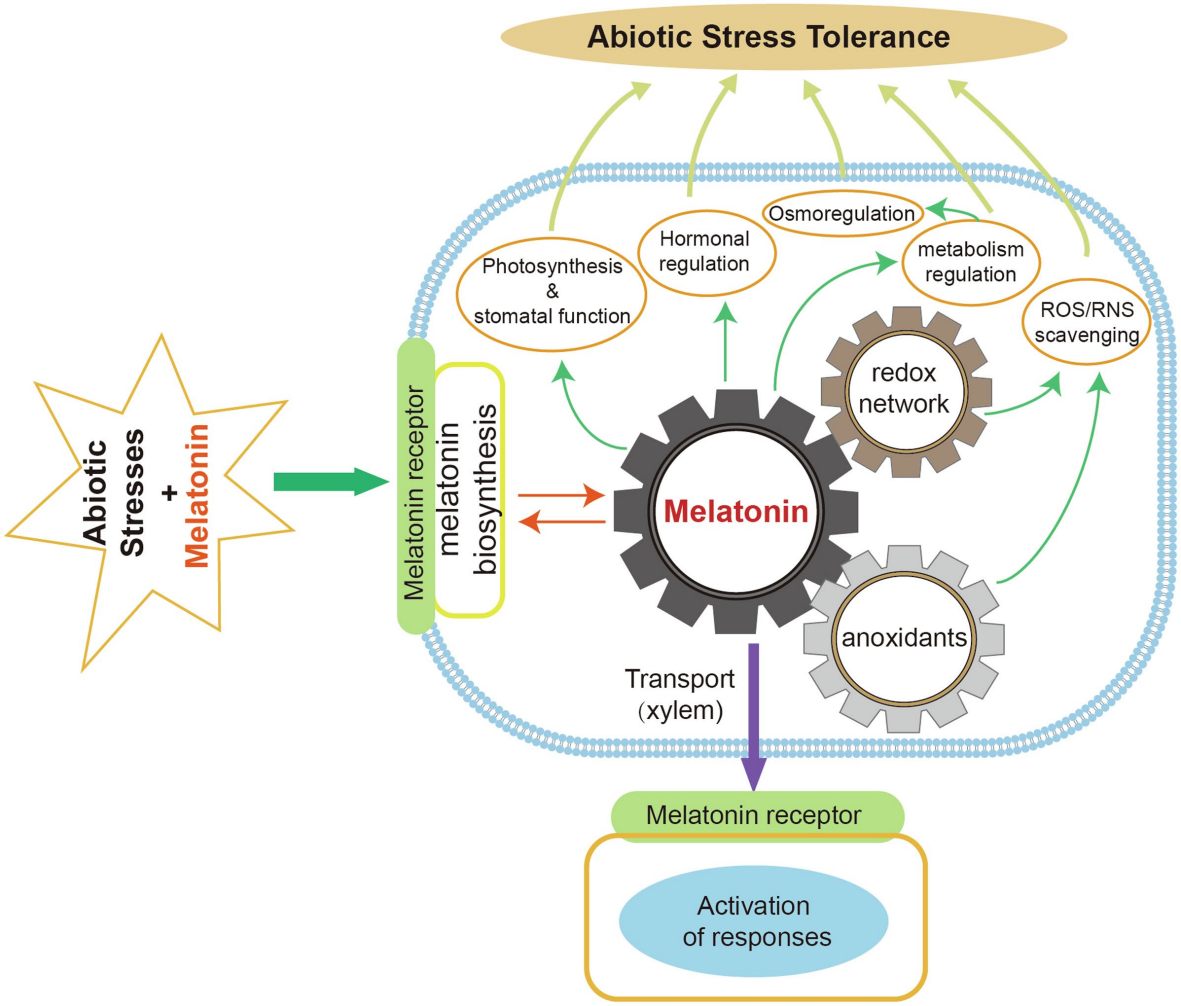
Roles of melatonin in improving plant abiotic stress tolerance. Exogenous melatonin can remove ROS and RNS by interacting with melatonin receptors to induce endogenous melatonin production and increased antioxidant levels. Melatonin facilitates photosynthesis and stomatal functions. It also regulates plant hormones, osmosis, and metabolism in response to abiotic stresses. Melatonin can be transported to various plant organs *via* the xylem and then activate stress responses.

The key melatonin biosynthetic enzymes (TDC, T5H, SNAT, ASMT, COMT) have been identified. The melatonin biosynthetic pathway has been characterized in several plant species. Under normal conditions, the chloroplast is the main biosynthetic site of plant melatonin. Mitochondria are also sites of melatonin biosynthesis in plants when the chloroplast pathway is blocked. The melatonin catabolic pathway and its metabolites have received increasing research attention in recent years because of their beneficial effects on plant resistance to environmental stresses. Exogenous melatonin application can regulate plant physiological and biochemical processes to enhance stress tolerance (Figure 5).

Several studies have demonstrated the powerful effects of exogenous melatonin on plant stress tolerance enhancement. However, the precise signaling, transcriptional, and epigenetic mechanisms of melatonin remain unclear and require further characterization. Although melatonin can influence the biosynthesis and signaling of other phytohormones, some mechanisms by which melatonin interacts with other phytohormones remain elusive. Furthermore, most melatonin-mediated stress tolerance studies have been performed using model plant systems, as well as some important crops. However, the transferability of these findings to other plant species should be investigated. Considering the role of melatonin in abiotic stress tolerance in plants, melatonin-related transgenic technologies could be developed to enhance stress resistance in plants.

Exogenous melatonin treatment has mainly been applied under laboratory-scale conditions. However, large-scale commercial and agricultural applications of melatonin have rarely been performed. Furthermore, the potential detrimental effects of melatonin treatment on plants grown under standard conditions must be investigated. Therefore, field studies are required to determine the effects of melatonin treatment on final crop yield and quality under regular conditions.

## Author Contributions

WZ and BJ: conceptualization. WZ and ZL: writing—original preparation. WZ, SM, and BJ: writing—review and editing. All authors contributed to the article and approved the submitted version.

## Conflict of Interest

The authors declare that the research was conducted in the absence of any commercial or financial relationships that could be construed as a potential conflict of interest.

## Publisher’s Note

All claims expressed in this article are solely those of the authors and do not necessarily represent those of their affiliated organizations, or those of the publisher, the editors and the reviewers. Any product that may be evaluated in this article, or claim that may be made by its manufacturer, is not guaranteed or endorsed by the publisher.
